# Pomalidomide improved immune profiles in myeloma

**DOI:** 10.18632/oncoscience.612

**Published:** 2025-01-14

**Authors:** Hannah Seah, Vaishnavi Reddy Bade, Lakshmi Bhavani Potluri, Srikanth Talluri, Rao H. Prabhala

**Affiliations:** ^1^Dana-Farber Cancer Institute, Boston, MA 02215, USA; ^2^VA Boston Healthcare System, Boston, MA 02130, USA; ^3^Harvard Medical School, Boston, MA 02115, USA

**Keywords:** myeloma, immune cells, pomalidomide

Multiple myeloma (MM) patients generally display worsening progression-free survival (PFS) upon successive relapse, with a potential cause being the loss of immunocompetency. In fact, the immune system is dysregulated in MM [1]. We observed a significant increase in CD4+ CD25+ T cells in patients with monoclonal gammopathy of undetermined significance (MGUS) and in patients with MM compared with healthy donors (25% and 26%, respectively, vs. 14%); however, T(reg) cells as measured by FOXP3 expression are significantly decreased in patients with MGUS and MM compared with healthy donors. Moreover, even when they are added in higher proportions, T(reg) cells in patients with MM and MGUS are unable to suppress anti-CD3-mediated T-cell proliferation in vitro assays systems [2]. We have shown here that T_h_17 cells are significantly elevated in peripheral blood and bone marrow of myeloma patients compared with healthy donors. Furthermore, when purified naive CD4 cells were cultured under T_h_17 polarizing conditions, T_h_17 cells were induced in significantly higher numbers in myeloma compared with healthy donors. Our results demonstrate that several T_h_17-associated cytokines, including IL-17, are significantly elevated in myeloma compared with healthy donors. In addition, we have shown that several other T_h_17-associated pro-inflammatory cytokines, including IL-1, IL-13, IL-17, and IL-23, are elevated after T_h_17 polarization in myeloma compared with healthy donors. We have further demonstrated by both ^3^H-thymidine incorporation and clonogenic assay that IL-17 increases myeloma cell proliferation. In addition, we have observed that IL-17 promotes myeloma tumor cell growth in SCID mouse model [3]. We observe significant inhibition of MM cell growth by anti-IL-17A monoclonal antibody both in the presence and the absence of BM stromal cells (BMSCs). More importantly, in the SCIDhu model of human myeloma administration of anti-IL-17A antibody weekly for 4 weeks after the first detection of tumor in mice led to a significant inhibition of tumor growth and reduced bone damage compared with isotype control mice [4].

The OPTIMISMM study for a large international study of MM patients (*n* = 540) with 1–3 prior lines of therapy, including previous lenalidomide relapsed/refractory patients, randomized to receive Velcade (V) and dexamethasone (d) with or without the addition of pomalidomide (P or POM) [5]. PVd treated patients displayed PFS of 20.7 months compared to 11.6 months for Vd treated patients, while the lenalidomide relapsed/refractory subset 17.7 and 9.5 months, respectively for PVd and Vd treatments. Immunomodulatory agents and proteasome inhibitors in combination with steroids are standard-of-care treatment in newly diagnosed patients but worsening prognoses with disease evolution have driven the development and approval of triplet regimens as a safe and effective standard of care in relapsed settings. It has been postulated that the clinical benefit of adding POM results from enhanced immunocompetency. POM targets Cereblon, which is part of the CRL4 E3 ubiquitin ligase, leading to the degradation of Ikaros and Aiolos, which are T cell repressors [6]. It has been reported that the pronounced increases in activated and proliferating NK and T cells, appreciably in CD8+ T cells, along with reduction in naive and expansion of effector memory compartments was seen in patients treated with pomalidomide along daratumumab, and dexamethasone. The frequency of CD4+ICOS+ cells in patients with greater than median, had favorable PFS of (21.9 months versus 15.9 months) for patients expressing lower than median [7]. In another trial, intermittent dosing of pomalidomide/dexamethasone in lenalidomide-refractory MM patients, activated NK and T cells frequencies are significantly elevated. Particularly, CD8+ T cells and NK cells showed higher expression of cytotoxic capacity with elevated levels of IFN-gamma, IL-2, Granzyme B and perforin following treatment and associated with clinical responses [8].

**Figure 1:**
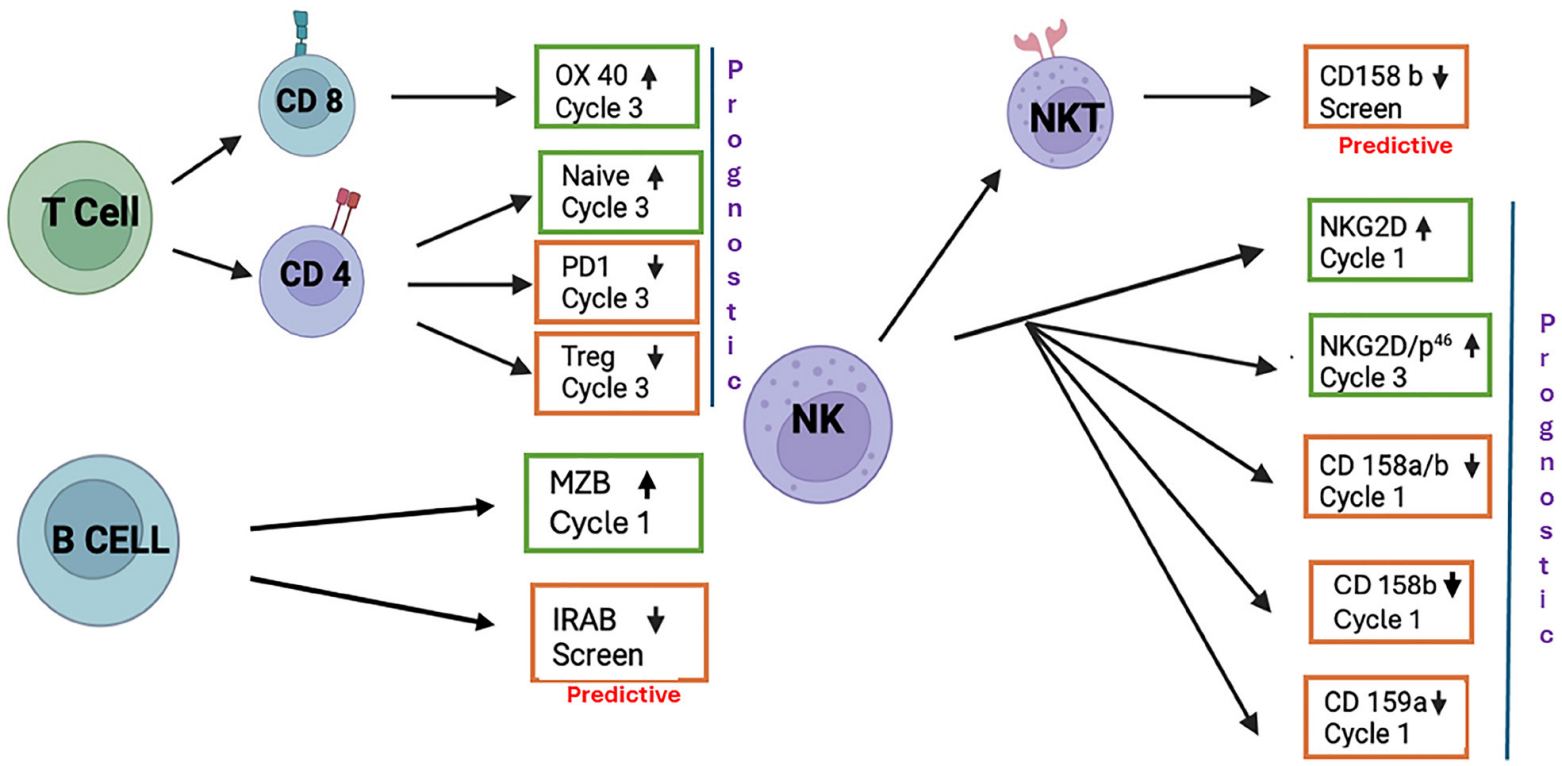
Pomalidomide-mediated immune changes associated with PFS. We have shown in three different immune cell-types, including T, B and NK cells, that the expression of a given immune marker in these cell-types is higher or lower than median level associated with better PFS in PVd treated patient population. We do not see this observation among Vd treated patients.

Here, we characterized the immune changes in relapsed/refractory MM patients (RRMM) treated with POM+velcade+dexamethasone (PVd) vs. velcade+dexamethasone (Vd) in the OPTIMISMM trial to understand the immunomodulatory effects of POM [9]. We assessed blood samples taken at three time points during treatment with flow cytometry to investigate immune subpopulations and surface markers. When comparing the two treatment arms, POM restored some immune cell subpopulations to counterbalance the immunosuppression in Vd-treated patients. The immune enhancements driven by POM in terms of its immune pharmacodynamics and immune profile changes by POM associated with PFS as shown in Figure 1, were observed in patients relapsing or refractory to a previous immunomodulatory agent treatment (lenalidomide). These results establish the immunostimulatory mechanisms of POM when combined with dexamethasone and velcade, likely contributing to its clinical benefits in RRMM.
